# Anatomical Characteristics of Duplicated Caudal Vena Cava in Cats—A Case Report

**DOI:** 10.3390/ani13101585

**Published:** 2023-05-09

**Authors:** Filip Korim, Mária Kuricová, Lada Eberlová

**Affiliations:** 1 Department of Morphological Disciplines, University of Veterinary Medicine and Pharmacy in Košice, 041 81 Košice, Slovakia; 2 Small Animal Clinic, University Veterinary Hospital, University of Veterinary Medicine and Pharmacy in Košice, 041 81 Košice, Slovakia; maria.kuricova@uvlf.sk; 3 Department of Anatomy, Faculty of Medicine, Charles University in Pilsen, 323 00 Pilsen, Czech Republic

**Keywords:** angiology, corrosion casting, development, felid, veins

## Abstract

**Simple Summary:**

The complete duplication of the caudal vena cava (CVC) in a 10-year-old male cat is presented based on a vascular corrosion cast. The literature search shows that views on the development of the pre-hepatic part of the CVC both in domestic mammals and of the inferior vena cava in humans vary considerably. Recent research using 3D reconstructions has brought new insight into the meaning of the caudal cardinal veins into this process. The highest incidence of this anomaly was found in rats, cats, and small breeds of dogs (3–27%), while in many more examined humans it was lower, estimated at 0.2–3.0%. In addition, the concomitant pathologies vary—in small domestic mammals the presence of a double CVC was often connected with the ureteral malposition, while in young adults the double inferior vena cava increased the risk of deep venous thrombosis. In 50% of bilateral venous thrombosis cases, coincidental congenital defects of the cardiovascular system were rare. We believe that the presented case and literature review can contribute to better knowledge regarding the deep abdominal veins—their development, variability, concomitant pathologies, and accurate diagnosis.

**Abstract:**

Precise knowledge of the species-/breed-specific anatomy is important for accurate diagnosis and treatment. Existing literature has also been increasing in accordance with the growing demands of biomedical research, wherein mammals, including cats, have been used worldwide. Based on a vascular corrosion cast, complete duplication of the caudal vena cava (dCVC) was accidentally found in a 10-year-old male cat. The two separate symmetric veins corresponding to two caudal venae cavae cranially directed on both sides of the aorta; their first tributaries were the duplicated right and left deep circumflex iliac veins, and the median sacral vein ended in the right common iliac vein. At the L4 vertebra level, the left caudal vena cava crossed the aorta ventrally. It united with the right CVC immediately above the renal veins at the level of the cranial mesenteric artery (L2–L3). Embryologic knowledge is essential to understand the differences between the CVC variants in domestic mammals and the inferior vena cava in humans. However, views regarding the post-hepatic segment of the CVC during development vary considerably. Therefore, our case report also includes a summary of the CVC developmental theories and their clinical impact. We believe that this case and literature review contribute to the knowledge regarding the deep abdominal veins’ variability, concomitant pathologies, and accurate diagnosis and surgery. Additionally, the latest robust studies demonstrating the exclusive participation of the caudal cardinal veins in the CVC development are discussed.

## 1. Introduction

Knowledge of species-/breed-specific anatomic variations is important not only for targeted veterinary care but also for the increased involvement of animal models in biomedical research [1]. Cats are used in undergraduate medical anatomy and biology classes because of their close resemblance to human anatomy. In addition to being cost effective, the use of cats in some countries helps to avoid the strict laws pertaining to human cadaver treatment [2].

**Figure 1:**
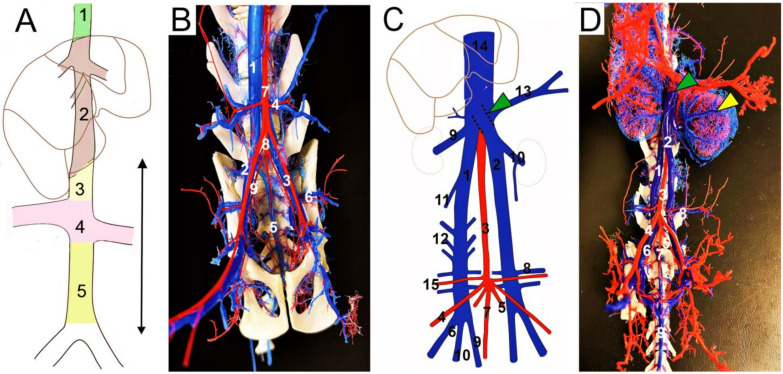
(**A**) Divisions of the caudal vena cava; (**B**) normal feline caudal vena cava (CVC); (**C**,**D**) duplicated CVC in cat—presented case; ventral view (**A**–**D**). (**A**) 1—post-hepatic segment of CVC; 2—hepatic segment of CVC; 3—cranial segment to the renal veins; 4—renal segment of CVC; 5—caudal segment to the renal veins; arrow—the pre-hepatic part of CVC. (**B**) 1—caudal vena cava; 2—right common iliac vein; 3—left external iliac artery and vein; 4—right deep circumflex iliac artery and vein; 5—median sacral artery and vein; 6—left iliolumbar artery and vein; 7—abdominal aorta; 8—common trunk of the internal iliac arteries; 9—right internal iliac artery. (**C**) 1—right caudal vena cava; 2—left caudal vena cava; 3—abdominal aorta; 4—right external iliac artery; 5—left internal iliac artery; 6—right external iliac vein; 7—median sacral artery; 8—left deep circumflex iliac vein; 9—median sacral vein; 10—left renal vein with left testicular vein; 11—right testicular vein; 12—lumbar veins; 13—left cranial abdominal vein; 14—caudal vena cava; 15—right deep circumflex iliac artery; green arrowhead—termination of the left caudal vena cava. (**D**) 1—right caudal vena cava; 2—left caudal vena cava; 3—abdominal aorta; 4—right external iliac artery; 5—left internal iliac artery; 6—right common iliac vein; 7—median sacral artery; 8—left deep circumflex iliac artery and vein; 9—median sacral vein; green arrowhead—termination of the left caudal vena cava; yellow arrowhead—capsular veins, left kidney.

Similar to the inferior vena cava (IVC) in humans, the normal caudal vena cava (CVC) is unpaired, and the pre-hepatic, hepatic, and post-hepatic parts can be clinically distinguished (Figure 1A). Concerning the different embryogenic origins, the CVC (IVC) is formed from a few segments, namely the pre-hepatic, hepatic, and post-hepatic segments (Figure 1A) [3,4]. The gross anatomy of the feline CVC is similar to that of other domestic mammals and is formed by the confluence of the right and left common iliac veins (Figure 1B) [5,6]. In cats, the site of the CVC origin is approximately at the level of the last lumbar vertebrae (L6–L7). The pre-renal segment runs in the midline, behind the aorta, and then passes ventrally and to the right [7]. The renal part of the CVC (level L2–L3) receives the right and left renal vein, and the right kidney with the right renal vessels are situated above their left counterparts in cats [5,6]. The pre-hepatic part of the CVC continues into the liver parenchyma into the hepatic segment and enters the *foramen venae cavae* in the tendinous center of the diaphragm. The terminal thoracic part passes ventrally to the caudal lobe of the right lung alongside the right phrenic nerve. The CVC terminates dorso-caudally in the right cardiac atrium [8].

Based on the results of retrospective veterinary studies on domestic animals, many CVC variants have been described [9], and their presence significantly increases the risk of accompanying ureteric anomalies or portosystemic shunts (Table 1). In the case that the collateral system provides an adequate venous return, most congenital venous anomalies, including duplicated CVC (dCVC), remain silent and asymptomatic [4,10,11]. Depending on the species/breed and the diagnostic device used, the prevalence of dCVC is 3–27% (Table 1) [4]. In both cats and dogs, the dCVC is associated with an increased incidence of a non-standard course of mainly the right-side ureters (Table 1). In contrast, the duplicated IVC (dIVC) prevalence in humans is lower, estimated at 0.2–3.0% [12,13,14]. Its presence increased the risk of deep venous thrombosis and concomitant developmental pathologies of the cardiovascular or renal systems [15,16,17].

Congenital anomalies are related to embryogenic development, which in the case of the mammalian CVC (corresponds to the IVC in humans) is considerably complicated and unclear, especially in the pre-/subhepatic part that includes the supra-/post-renal, renal, and infra-/pre-renal segments. The discrepancies between different developmental theories could be attributed to several factors, including different methodologies and inconsistencies in the nomenclature [3,4,10,18,19,20,21,22,23,24,25,26,27,28].

Based on the vascular corrosion cast and comparative anatomy, we present a case of a dCVC found accidentally in an adult domestic cat and review the available literature of dCVC (dIVC in humans) development, prevalence, and clinical impact. Comparative anatomy is fundamentally important in biomedicine when planning experiments or translating research results to human medicine. In addition to the known anatomical or developmental differences, inconsistencies in translating veterinary medicine to human medicine could also originate from the terminology differences between veterinary and human medicine. The terminology used in this study is based on the current standardized nomenclature [29,30].

**Table 1:** Review of the caudal vena cava anomalies in cats, dogs, and guinea pigs.

Species	Sex	Diagnostic Method	Number	Prevalence of dCVC Anomaly	Prevalence of Accompanied Pathologies	References
Domestic cat	Not specified	Necropsy	301	dCVC in 7%, of which 21% were accompanied with ureteric anomalies	Circumcaval/retrocaval ureter in 32%, of which 80% were affected on the right side	Bélanger et al., 2014 [31]
Domestic cat	50.4% females and 49.6% males (90.4% castrated)	CT angiography, USG, and MRI	272	dCVC in 5.8%	Circumcaval/retrocaval ureter in 12.5%, of which 87.5% had dCVC	Pey et al., 2015 [23]
Domestic cat	Female	Necropsy	1	dCVC	Left retrocaval ureter	Casteleyn et al., 2015 [21]
Domestic cat	Female	Necropsy	1	dCVC	Circumcaval ureters and diaphragmatic hernia	Chisco et al., 2016 [25]
Domestic cat	Female	Necropsy	1	dCVC	None	Stocco et al., 2019 [28]
Domestic dog	Not specified	CT and USG	7913	Prevalence of dCVC was 2.08% on CT and 0.46% on USG; prevalence was significantly higher in small breeds than in large breeds	Extrahepatic portosystemic shunts	Bertolini et al., 2014 [4]
Domestic dog	Not specified	CT	121	CVC split in 14% (99% affected on the right side): partial duplication in 7% and complete duplication in 6%	Not specified	Ryu et al., 2019 [9]
Guinea pig	50 males and 50 females	Embalming, observation of transverse sections	100	dCVC in 54% (30% in males and 24% in females)	None	Nakamura et al., 2019 [32]

CT—computed tomography, CVC—caudal vena cava, dCVC—duplicated caudal vena cava, MR—magnetic resonance, USG—ultrasound.

## 2. Materials and Methods

The random carcass of a mixed-breed, intact male cat (*Felis catus*) was used in this study. The cat was 10 years old and weighed 4.7 kg. The cadaver was used during educational processes at the Small Animal Clinic.

To remove the blood remains, the entire carcass was perfused through the ascending aorta with 0.9% sodium chloride solution (sodium chloride, Mikrochem Trade, s.r.o., Pezinok, Slovakia) and 4.2% sodium citrate (Tri-sodium citrate dihydrate, Mikrochem Trade, s.r.o., Pezinok, Slovakia). Following this, acrylic self-curing dental resin Duracryl^®^ Plus (SpofaDental a.s., Jičín, Czech Republic) was administered through the ascending aorta (resin dyed red) and through the caudal vena cava (resin dyed blue). After resin polymerization, corrosion of the soft tissues was performed in a maceration unit (BM 1115, Gastrolux, s.r.o., Žilina, Slovakia) using 2% sodium hydroxide (sodium hydroxide, Mikrochem Trade, s.r.o., Pezinok, Slovakia) for 3 days at 70 °C [33]. After removal of the soft tissue, the cast was washed with tap water, dried at 22 °C, and inspected macroscopically and microscopically using a surgical microscope (Carl Zeiss Movena S7, Carl Zeiss AG, Oberkochen, Germany).

Measurements were performed using a Proteco digital calliper (Proteco Náradie, s.r.o., Bratislava, Slovakia) with an accuracy of 0.01 mm.

## 3. Results

The casting procedure enabled evaluation of only the skeleton and the casted vascular structures (Figure 1B,D). The finding was the abnormal formation of the CVC with two separate, symmetric right and left caudal venae cavae joined together between L2 and L3, under the caudate process of the liver, immediately above the renal vein termination, and 5 mm caudal to the origin of the cranial mesenteric artery (Figure 1C,D). Both caudal venae cavae originated at the L6–L7 level by the union of the external and internal iliac veins. The right caudal vena cava (RCVC) extended on the right side of the abdominal aorta, collecting the right cranial abdominal vein (*v. abdominalis cranialis*), right renal vein (*v. renalis dextra*), both right and left lumbar veins (*vv. lumbales*), and duplicated right deep circumflex iliac veins (*v. circumflexa ilium profunda*). The left caudal vena cava (LCVC) ran along the left side of the abdominal aorta, collecting the left cranial abdominal vein, left renal vein, and duplicated left deep circumflex iliac vein (Figure 1C,D). At the L4 level, it crossed over the aorta and routed to the point of confluence; the diameters of the LCVC and RCVC were 3.7 mm and 3.8 mm, respectively. The median sacral vein (*v. sacralis mediana*) joined to the right common iliac vein. The portal vein was not cast. The origins and courses of the other investigated abdominal vessels corresponded to the normal anatomical layout, and only few local cast defects were observed.

## 4. Discussion

### 4.1. Pre-Hepatic Caudal Vena Cava in Domestic Mammals and Infrahepatic Human Inferior Vena Cava Development

The normal CVC in domestic mammals converts to a unilateral, mainly right-sided vein. In cats, it is formed by the union of the right and left common iliac veins at the level of the last lumbar vertebra, with each vein starting at the confluence of the external and internal iliac veins. The feline CVC receives the paired lumbar, renal, and phrenic veins and the unpaired hepatic, right adrenolumbar, and right gonadal veins [5,6,7].

**Figure 2:**
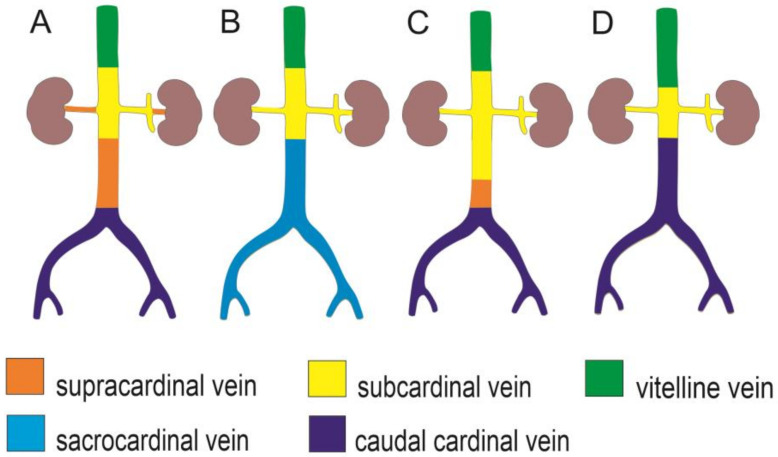
Different theories of the caudal (inferior) vena cava development, see also Table 2. The main portion of the caudal segment to the renal veins is formed by the supracardinal veins (**A**), the sacrocardinal veins (**B**), the subcardinal veins (**C**), or by the caudal cardinal veins (**D**).

Based on a literature review, the existing knowledge regarding mammalian CVC and human IVC development is controversial, especially for the pre-/post-hepatic segments [24]. This may be due to species-specific diversity, technical artefacts, biased interpretations, and/or inconsistent terminology [3,34,35]. It is widely accepted that the IVC development occurs within the Carnegie stages (CS) 11–23. Moreover, it follows a strict temporospatial arrangement involving partial fusion, regression, and anastomosis between the paired venous precursors and their sprouts, namely the posterior cardinal, supracardinal, subcardinal, caudal cardinal, and/or lateral sympathetic veins (Figure 2, Table 2) [3,22,24,34]. During development, the left venous channels regress and a single right-sided CVC (IVC) first appears in the segments cranial to the metanephros. Regarding the CVC segment caudal to the renal veins (i.e., the pre-renal segment) and azygos system, two main views have prevailed in the current human embryology textbooks (Figure 2, Table 2). One is that the supracardinal veins contribute to the origin of both the most caudal CVC (IVC) and azygos veins (Figure 2, Table 2) [36]; the second is that the sacrocardinal veins are partly involved in the origin of the caudal segments of the CVC (IVC) and iliac veins (Figure 2B, Table 2) [37]. These views were repetitively studied and questioned by Cornillie and Simoens (2005 and 2008) [3,34] and Hikspoors et al. (2016) [24] using human, pig, and rodent embryos [3,24,33]. Their studies based on three-dimensional embryo reconstructions compared the IVC development in species with different degrees of mesonephric development and clearly demonstrated that in all these species, the segment of the CVC caudal to the renal veins developed from the right caudal cardinal vein (CCV) only and the renal part from the subcardinal veins. The subcardinal veins developed from the CCV, irrespective of the degree of mesonephric development. Based on the 3D reconstructions and in accordance with the two functional portal venous entities, the authors were inclined to retain and distinguish two developmental venous systems: the CCVs for the supply and the subcardinal veins for the drainage of the organs of the urogenital ridge.

### 4.2. Differences between the Duplicated Caudal Vena Cava and Inferior Vena Cava

In domestic mammals, the prevalence of congenital anomalies of the CVC is 3–27% (Table 1) [4,9]. This could be related to the length, course, quantity (duplication or agenesis), residual remnants, anastomoses, or abnormal lumen width [38]. Regarding the dCVC, the following two anomalies have been distinguished: complete duplication, which includes the renal segments, and partial duplication, which only involves the pre-renal segment [4]. The paired CVC is a standard anatomical structure in marine mammals, such as dolphins and whales [20]. As an anomaly, the variably occurring dCVC (dIVC) has been repeatedly reported in dogs, cats, and guinea pigs, as well as in humans (Table 1) [10,11,28,32,39]. However, the prevalence and concomitant pathologies seem to be species-/breed-specific. The dCVC and atypical porto-caval anastomoses were found commonly in small dog breeds [27]. Other studies on domestic mammals have shown an increased co-occurrence of the dCVC and malpositioned ureter [4,21], and the pre-ureteral vena cava rarely resulted in an increased risk of concurrent urinary signs [9]. No sex-related differences were observed. In humans, the dIVC prevalence is lower, estimated to be 0.2–4% [16,20]; the anomaly was associated with cardiovascular defects, and it was only sporadically coincidental with anomalous ureter or renal agenesis [17]. In contrast, the risk of the deep venous thrombosis increased significantly with dIVC in young men [16].

### 4.3. Duplicated Caudal Vena Cava in Cats and Dogs

Several descriptive studies focusing on the dCVC have also been conducted in cats (Table 1). Bélanger et al. (2014) collected data of 574 cats. The prevalence of dCVC was 7%, with each CVC draining the ipsilateral renal vein. The ureteric anomaly was present in 21% of the cases, of which 18% had retrocaval ureters and 80% had right-sided ureters. The association between retrocaval ureter and dCVC was confirmed in 4% of cases, and 95% of the animals with dCVC had circumcaval ureters. None of the cats (n = 106) with circumcaval ureters showed any clinical signs of urinary infection or dilatation. Moreover, no sex predisposition was observed [31]. Pey et al. (2015) determined the presence of dCVC in 16 of 272 cats (6%), and concomitant retrocaval ureters occurred in 80% of the cats [23]. Stocco et al. (2019) described the occurrence of dCVC in a Brazilian shorthaired cat, wherein two asymmetric CVCs were found with the left CVC receiving the ipsilateral gonadal and renal veins. The confluence with the right CVC occurred at the level of the left adrenal gland, but the vertebral level was not specified. Notably, the authors also reported a communicating branch between the two CVCs [28]. In our case, the level of confluence of the two symmetric right and left venae cavae occurred between L2 and L3, the left CVC crossed the aorta ventrally, and no transverse CVC anastomosis was found. Both the right and left lumbar veins terminated in the right CVC (Figure 1C,D).

The sensitivity of different diagnostic devices was studied by performing ultrasonography (USG) and CT in a large population of dogs (n = 3407) by Bertolini et al. (2014) [4]. The prevalence of dCVC varied from 0.4% confirmed on USG to 2% on CT. Additionally, a high risk of dCVC incidence was evident in some small and low-weight breeds of dogs, such as Maltese dogs, Poodles, Yorkshire terriers, and West Highland white terriers. In their study, all the circumaural ureters were right-sided and asymptomatic. Moreover, concomitant anomalous portosystemic shunts and dCVCs were more commonly seen in small and toy breeds of dogs, such as Maltese dogs and Yorkshire terriers, in the study by Tobias and Rohrbach (2003) [40].

### 4.4. Clinical Relevance

An increased risk of venous stasis and thrombosis has been observed in humans with CVC anomalies [16,39,41]. Thrombosis and stasis can be caused by insufficient retrograde blood flow and elevated blood pressure in the lower extremities [16,39]. In terms of perfusion in dogs with dCVC, the venous blood flow showed normal velocity in both CVCs [4]. It was hypothesized that quadrupedal walking and a typical dog’s locomotion pattern may play a role in preventing thrombosis and venous stasis. Neither venous thrombosis nor venous stasis was detected in cats (Table 1). Planned abdominal surgeries for spaying or neutering pets usually involve the urogenital system [42]. The preoperative veterinary diagnostics of abdominal pathologies have dramatically improved and mostly correspond to the perioperative findings; however, major discrepancies (25%) were observed between the USG findings and perioperative surgical findings in cases of malignancies [43].

Early identification of such anomalies is important for abdominal and pelvic/retroperitoneal surgical interventions also in human medicine, primarily for the IVC filter placement. The presence of the left IVC can be misdiagnosed as adenopathy, especially if the contrast enhancement of the vein is poor due to technical reasons. Additionally, recurrent pulmonary emboli despite anticoagulation therapy should raise the suspicion of dIVC. In young adults with CVC anomalies, deep venous thrombosis is bilateral in more than 50% of the patients [39]. If present, the retrocaval ureter, also known as the circumcaval ureter, can be significantly compressed, resulting in hydronephrosis or recurrent urinary tract infections [39].

Even unique coincidental anomalies may indicate developmental interactions and synchronicity and contribute to their understanding. For instance, based on the CT scans, Vignesh and Bhat (2022) diagnosed duplicated superior vena cava and dIVC in a patient with left renal agenesis [44]. We believe that accidental necropsy or casting findings may have similar benefits.

## 5. Conclusions

Congenital anomalies of the CVC vary and are quite common in small mammals; hence, their understanding is essential for precise clinical diagnosis and interventions. In our case, the finding of the complete dCVC was based on a vascular corrosion cast of a domestic cat. The point of confluence of the two symmetric right and left venae cavae was above the renal veins at L2–L3, and neither an atypical ureter nor transverse venous interconnection was detected. Based on the literature search, the possible co-occurrence of ureteric anomalies and dCVC should be considered. Notably, the dCVC increases the risk of bilateral deep venous thrombosis in young adults. These findings may be essential for both differential diagnosis and reducing the risk of perioperative complications, especially in oncological surgeries or IVC filter placement.

Regarding the IVC embryology, within the last 15 years, 3D mammal embryo reconstructions repeatedly proved the development of the CVC exclusively from the CCV. Additionally, inconsistent developmental theories were elucidated on the topographical base. Considering the robustness of these studies and the persuasiveness of their results, we believe that the hypothesis of the CVC developing from the CCV should be reconsidered by the professional community and literature.

## Figures and Tables

**Table 2:** Theories regarding the infrahepatic caudal vena cava or inferior vena cava precursors in domestic mammals and humans (Cornillie and Simoens, 2005; Hikspoors et al., 2015) [3,22].

IVC/CVC Segment	Supracardinal Model (Huntington and McClure, 1920) [18]	Caudal Cardinal Model (Butler, 1927; Hikspoors et al., 2015) [19,22]	Sacrocardinal Model (Sadler, 2011) [37]
Renal	Right supracardinal vein, supracardinal-subcardinal anastomosis	Subcardinal veins	Subcardinal veins
Infrarenal/Pre-renal	Right supracardinal vein	Right caudal cardinal vein	Right sacrocardinal vein
Confluence of the common iliac veins	Posterior cardinal veins		
Duplicated CVC precursor	Persistent left supracardinal vein (Bertolini et al., 2014) [4]		Persistent intersubcardinal anastomosis (Sadler, 2011) [37]

CVC—caudal vena cava, IVC—inferior vena cava.

## Data Availability

Data are published in this article.
